# Supplementary figures and images for: Genome-wide association study of yield components in spring wheat collection harvested under two water regimes in Northern Kazakhstan
Source: PeerJ. 2021 Jul 27;9:e11857. doi: 10.7717/peerj.11857 (PMC8323601; doi:10.7717/peerj.11857)

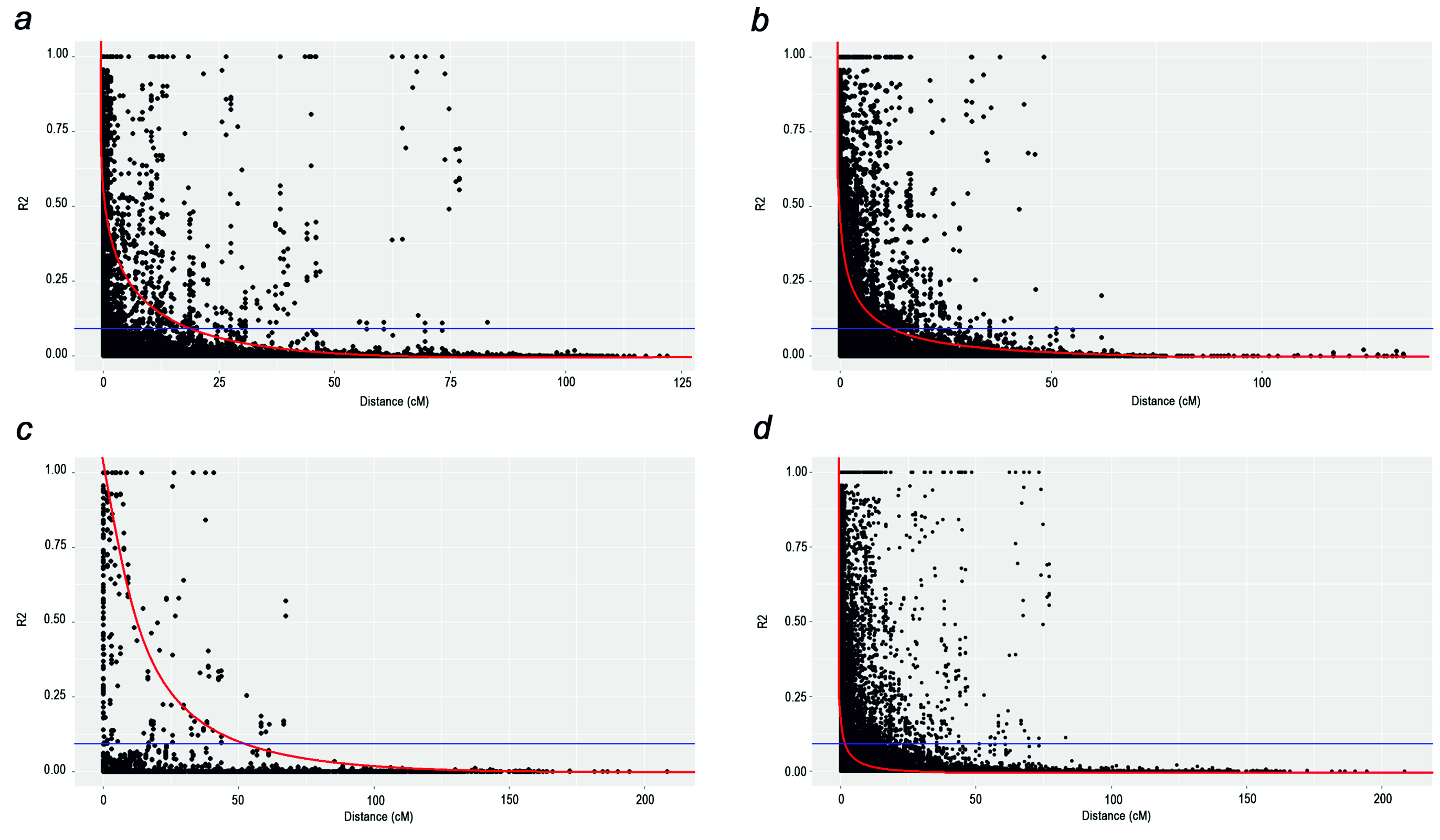

Supplement: Supplemental Information 3 — Note: red line –LD decay curve, blue line - R2 threshold, R2 = 0.1. [file peerj-09-11857-s003.jpg]

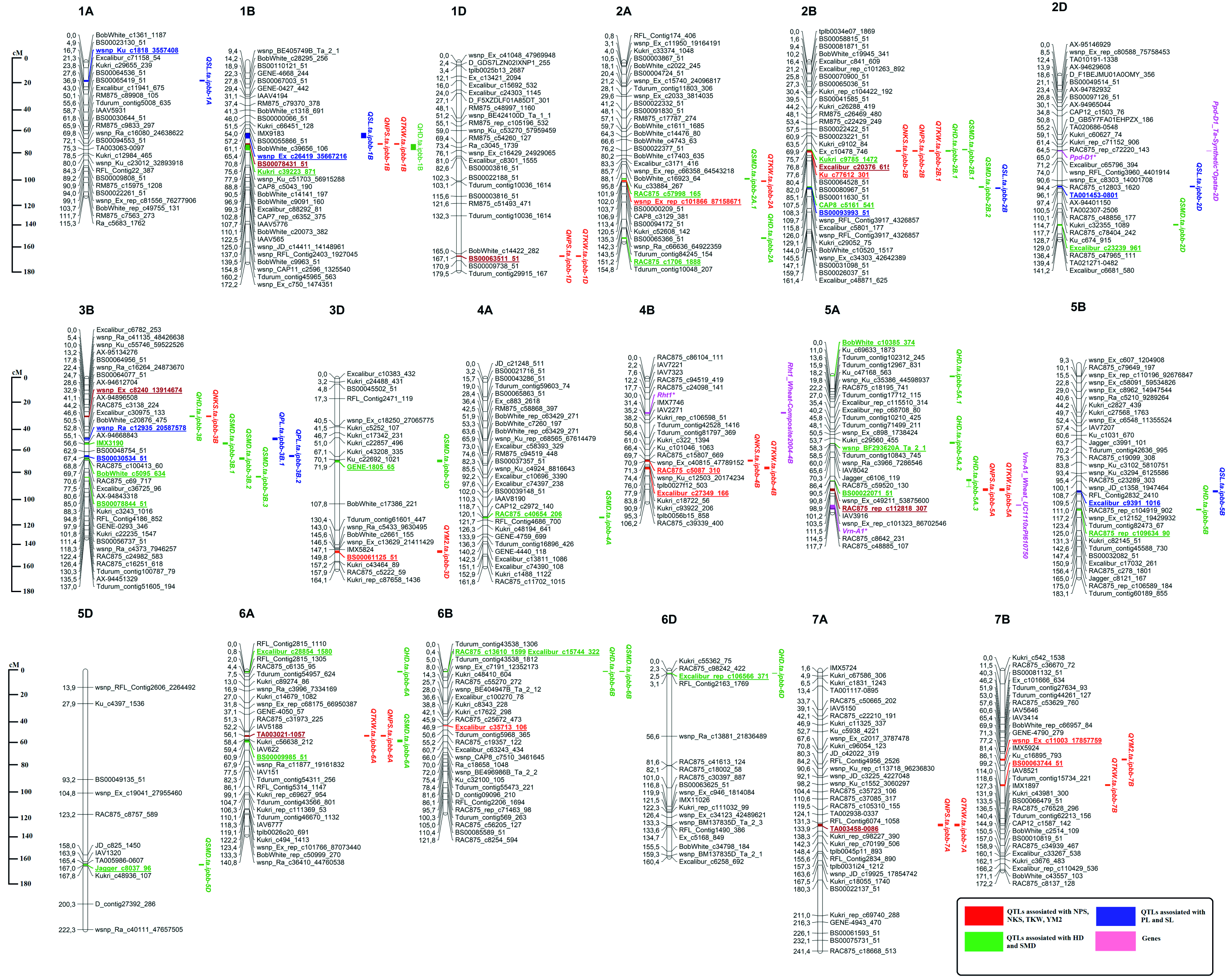

Supplement: Supplemental Information 4 — The markers names are shown on the right and positions of marker loci are shown on the left of the linkage maps in centimorgans (cM). Significant markers, the identified QTLs, red for traits NKS, TKW, YM2, blue for PL and SL traits , green for HD and SMD, and pink for genes. [file peerj-09-11857-s004.jpg]
